# Functionalized Electrospun Nanofibers as Colorimetric Sensory Probe for Mercury Detection: A Review

**DOI:** 10.3390/s19214763

**Published:** 2019-11-02

**Authors:** Brabu Balusamy, Anitha Senthamizhan, Tamer Uyar

**Affiliations:** 1 Fondazione Istituto Italiano di Tecnologia, Via Morego, 30, 16163 Genova, Italy; drbrabu@gmail.com (B.B.); dranitha35@gmail.com (A.S.); 2 Department of Fiber Science & Apparel Design, College of Human Ecology, Cornell University, Ithaca, NY 14853, USA

**Keywords:** mercury, sensor, electrospinning, nanofiber, colorimetric, fluorescence, functionalization, direct incorporation, surface decoration

## Abstract

Mercury is considered the most hazardous pollutant of aquatic resources; it exerts numerous adverse effects on environmental and human health. To date, significant progress has been made in employing a variety of nanomaterials for the colorimetric detection of mercury ions. Electrospun nanofibers exhibit several beneficial features, including a large surface area, porous nature, and easy functionalization; thus, providing several opportunities to encapsulate a variety of functional materials for sensing applications with enhanced sensitivity and selectivity, and a fast response. In this review, several examples of electrospun nanofiber-based sensing platforms devised by utilizing the two foremost approaches, namely, direct incorporation and surface decoration envisioned for detection of mercury ions are provided. We believe these examples provide sufficient evidence for the potential use and progress of electrospun nanofibers toward colorimetric sensing of mercury ions. Furthermore, the summary of the review is focused on providing an insight into the future directions of designing electrospun nanofiber-based, metal ion colorimetric sensors for practical applications.

## 1. Introduction

Heavy metal pollution of water resources poses a major threat to human and environmental health because of the associated risks. Although several efforts have been made, rapid urbanization and anthropogenic activities have led to a continuous increase in heavy metal pollution in water resources, and thus, concentrations are not within the recommended limits of various global regulatory authorities. Therefore, intensive attention needs to be paid to monitoring the levels of heavy metals, including arsenic, mercury, chromium, nickel, and cadmium, to minimize the associated adverse health effects resulting from bioaccumulation and biomagnification [1,2,3,4,5]. Among the various heavy metals, mercury is considered the most toxic element, owing to its severe effects, including cardiovascular disease, nephrotoxicity, neurotoxicity, carcinogenicity, immunotoxicity, and reproductive and developmental toxicity [6,7,8,9,10]. The ecological and toxicological effects of mercury are mainly influenced by its chemical forms, as the natural physical, chemical, and biochemical processes in the aquatic environment transform mercury into different inorganic and organic forms. Specifically, lower organisms are capable of transforming inorganic mercury into methylmercury, which is a potential neurotoxicant [11,12,13].

Therefore, detecting mercury ions in the aquatic environment is of significant concern. Accordingly, several approaches have been employed in the detection of mercury ions, including inductively-coupled plasma mass spectrometry, surface-enhanced Raman spectroscopy, and atomic absorption spectrometry, which are costly and require time-consuming pre-treatment and people with specialized training for operation [14,15,16]. In the recent past, colorimetric sensors attracted tremendous interest for the detection of mercury ions because of their miniaturization and potential for utilization in field applications. In this context, fluorescent, nanomaterial-based colorimetric sensors with various functionalization approaches have made a considerable impact on mercury ion detection [17,18,19,20,21,22]. However, the majority of the investigations are based on the sensing of mercury ions in the solution phase. The practical and real-time applications prefer solid-state sensing methodologies, owing to their easy handling, facile operation, simplicity, stability, and portability.

**Figure 1:**
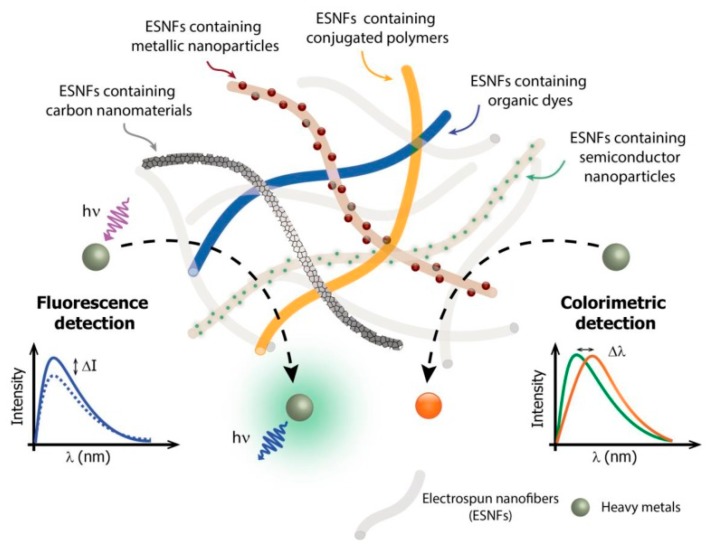
Scheme of electrospun nanofibers (ESNFs) modified by distinct nanomaterials for applications in optical sensors for heavy metal detection. The lower part of the figure provides a schematic showing the principles of optical detection by the fluorescence quenching (left) or by the color change (right) of the ESNFs in the presence of a heavy metal ion. Reprinted from [37], 2017, MDPI.

Electrospun nanofibers offer promising characteristics, including nanoscale fiber diameters, a large surface area, a nanoporous structure, design flexibility and functionalization with various molecules through different approaches, extreme flexibility, proper mechanical strength, and easy fabrication from a wide range of materials with diverse structural features, which extends their potential in various sectors from environmental to biomedical applications [23,24,25,26,27,28,29,30,31,32,33,34,35]. The electrospun nanofibers especially, have numerous potential methods for the functionalization of various active agents for the selective and sensitive detection of target agents, turning them into an excellent, solid support platform for sensing. Therefore, recent years have shown several approaches in the fabrication of electrospun nanofibers in the detection of heavy metals [36,37]. Figure 1 briefly demonstrates the various materials used for the functionalization of electrospun nanofibers and their heavy metal sensing mechanism [37]. The most significant advantage of colorimetric sensing is the visually-noticeable color change following exposure to heavy metals, which is a remarkable characteristic needed for real-time field applications. In this review, we highlight recent advances in fabricating electrospun nanofiber platform-based colorimetric sensors through direct incorporation and surface decoration approaches for the detection of mercury ions (Hg^2+^) with representative examples. Further, we summarize the review, and a future direction for the development of colorimetric sensors is discussed.

## 2. Functionalized, Electrospun Nanofibers for Colorimetric Mercury Ion Detection

### 2.1. Direct Incorporation

**Figure 2:**
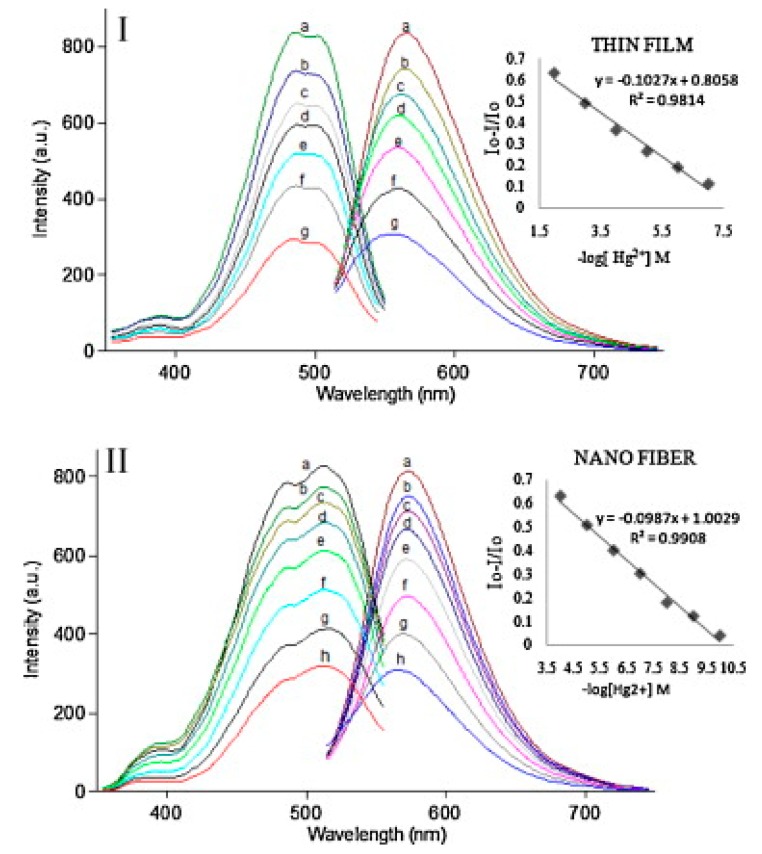
**(I)** Fluorescence response of the DC-AZM-doped, EC-based thin film to Hg (II) ions at pH 4.0. (a) Hg-free buffer, (b) 10^−7^, (c) 10^−6^, (d) 10^−5^, (e) 10^−4^, (f) 10^−3^, and (g) 10^−2^ M Hg (II). Inset: calibration plot for the concentration range of 10^−7^–10^−2^ M Hg (II). (**II**) Response of the EC based nanofiber to Hg (II) ions at pH 4.0. (a) Hg-free buffer, (b) 10^−10^, (c) 10^−9^, (d) 10^−8^, (e) 10^−7^, (f) 10^−6^, (g) 10^−5^, and (h) 10^−4^ M Hg (II). Inset: linearized calibration plot for the concentration range of 10^−10^–10^−4^ M Hg (II). Reprinted with permission from [39]. Copyright 2012, Elsevier.

To date, there has been tremendous progress made in the design and fabrication of nanofibers through the blending of inorganic/organic functional materials with polymeric materials followed by electrospinning to enhance the functional properties for specific applications. The resultant nanofibers possess advantageous features, including being stable, flexible, lightweight, and mechanically strong from the organic polymers, while the incorporation of functional nanomaterials achieves the functionality. Such electrospun nanofibers have shown tremendous applicability in the energy, filter, sensor, and biomedicine sectors [38]. Therefore, this approach can also be extended to fabricate the electrospun nanofibers through blending functional materials that are capable of detecting Hg^2+^ ions with a polymeric matrix, which is believed to preserve the functional characteristics. To date, several functional materials have been incorporated in electrospun nanofibers for the sake of detecting mercury ions. In this context, Kacmaz et al. have demonstrated the fabrication of ethyl cellulose (EC)-based electrospun nanofibers for the sub nanomolar optical detection of the ionic mercury by using azomethine ionophore [39]. Briefly, the electrospinning precursor solution was optimized through varying concentrations of EC polymer, plasticizer (dioctyl phthalate), room temperature ionic liquid (RTIL, 1-ethyl-3-methylimidazolium tetrafluoroborate (EMIMBF4)), and (4-(dimethylamino) benzaldehyde2-[[4-cyanophenyl]methylene]hydrazine (DC-AZM) dye. Furthermore, the precursor solution mixture was electrospun to obtain the composite nanofibrous membrane and was applied for the detection of mercury ions. Owing to the high ionic conductivity, non-volatile nature, low toxicity, and chemical and thermal stability, the EMIMBF4 was selected as a polymer electrolyte. Additionally, a thin film with a similar composition to the electrospun nanofibrous membrane was fabricated for a comparison of detection performance. The DC-AZM dye turned the Hg(II) ions into selective probes after the doping of anionic additive potassium tetrakis-(4-chlorophenyl) borate in EC matrices. The anionic additive plays a critical role in the extraction of Hg(II) ions into the polymer phase; meanwhile, potassium ions diffuse into the aqueous phase from the nanofibrous membrane thanks to the ion-exchange phenomenon, owing to electroneutrality. The comparative response of the thin film and EC-based nanofibers to various concentrations of Hg (II) ions are illustrated in Figure 2. The Hg (II) ions are responsible for the DC-AZM dye fluorescence intensity changes noted at 572 nm due to the collision and complex formation. The nanofiber-based sensor has shown an enhanced response to Hg (II) ions compared to the thin film prepared using a similar composition, and the limit of detection was found to be 0.07 nM. The high sensitivity observed may be associated with the higher surface area and the diffusion of mercury ions to the ionophores. The response of nanofiber-based sensor has proven it an alternative technique to the cold-vapor atomic absorption spectrometry (CV-AAS), inductively coupled plasma-mass spectrometry (ICP-MS), and flame emission methods.

**Figure 3:**
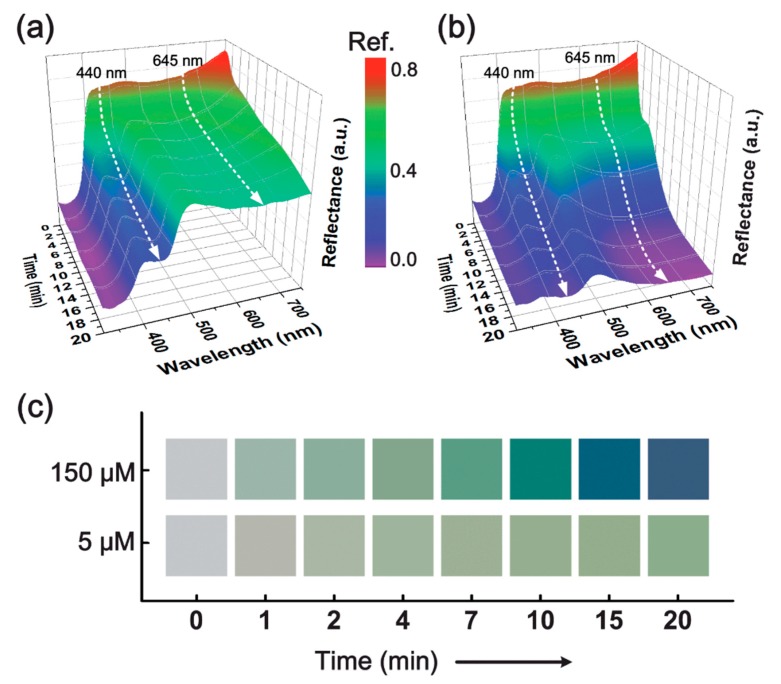
Kinetic reflectance response of the polyaniline-leucoemeraldine base nanofiber (PANI-LBNF) sensor strips as a function of time for different Hg^2+^ concentrations: (**a**) 5 μM and (**b**) 150 μM. (**c**) The corresponding time-dependent visualization of CIELAB color changes versus Hg^2+^ concentration. Reprinted with permission from [40]. Copyright 2014, The Royal Society of Chemistry.

Hierarchically-nanostructured, conjugated polymers have been employed in the preparation of an electrospun nanofiber-based sensor strip for the colorimetric detection of Hg^2+^ ions [40]. Concisely, a polyaniline (PANI)-based sensor was fabricated for the practical, simple, and high-throughput colorimetric determination of trace levels of Hg^2+^ ions using the combination of electrospinning and an in situ reaction. PANI is a well-explored, conjugated polymer, which possesses several unique characteristics, including electrical conductivity, good redox reversibility, and environmental stability. Based on ratio between the reduced benzenoid amine to the oxidized quinoid imine, the oxidation state of PANI may vary between leucoemeraldine base (PANI-LB), emeraldine base (PANI-EB), and pernigraniline base (PANI-PB) with respect to its fully reduced, half oxidized, and fully oxidized states, respectively. Notably, the number of amine and imine functional groups in PANI have strong influences in the interaction/complexation with Hg^2+^ ions. In a typical experimental process, PANI emeraldine salt was synthesized through the chemical polymerization of aniline. For the purpose of electrospinning, the resultant PANI emeraldine salt was further processed to attain the PANI-EB powder. The electrospinning precursor solution was prepared by dissolving the PANI-EB, polyvinylbutyral (PVB), and polyamide-6 (PA-6). Afterward, the electrospinning was performed to get the PANI-EBNF composite nanofibrous membrane, which subsequently underwent an in situ reduction reaction using a treatment with hydrazine. The resultant membrane was washed and dried for obtaining the PANI-LBNF membranes that were further used for the sensing measurements. The sensor strip in this investigation was designed based on three criteria, (1) the PANI probes must possess a specific optical response to Hg^2+^, (2) the PANI probes should homogeneously immobilize into the substrate, and (3) the sensing nanofiber membrane has hydrophilicity, porosity, and stability in water. The PANI-LB has the potential to instigate “turn-on” and “color-change” following exposure with Hg^2+^ ions. PVB and PA-6 were chosen as template materials as they provide good electrospinnability, membrane strength, and miscibility with PANI. The resultant sensing nanofiber membrane was systematically characterized for morphology, the presence of functional groups, and porous nature. The colorimetric sensing experiment was performed by exposing the sensor strip to various concentrations of Hg^2+^ ions ranging from 0.5 nM to 150 μM for 20 min. The corresponding results revealed distinguishable color changes from white-yellow to green-blue with a visual detection limit of 5 nM. Furthermore, the kinetic sensing response of the sensor strips was studied through continuous monitoring of the reflectance spectra, following exposure to Hg^2+^ as a function of time, and the outcome is presented in Figure 3. As can be seen from Figure 3a, the reflectance intensity of 440 nm gradually decreased over time, whereas the intensity at 645 nm was found to be relatively stable (>7 min) in the case of Hg^2+^ exposure at 5 μM. On the other hand, exposure of 150 μM resulted in rapid decreases of spectral intensity between 0–15 min time period (Figure 3b,c), suggesting complete oxidation of benzenoid segments into fully oxidized PANI-PB; hence, resulting in the vivid blue color. Furthermore, the sensors displayed good selectivity over a variety of cations and reversibility following regeneration cycles.

**Figure 4:**
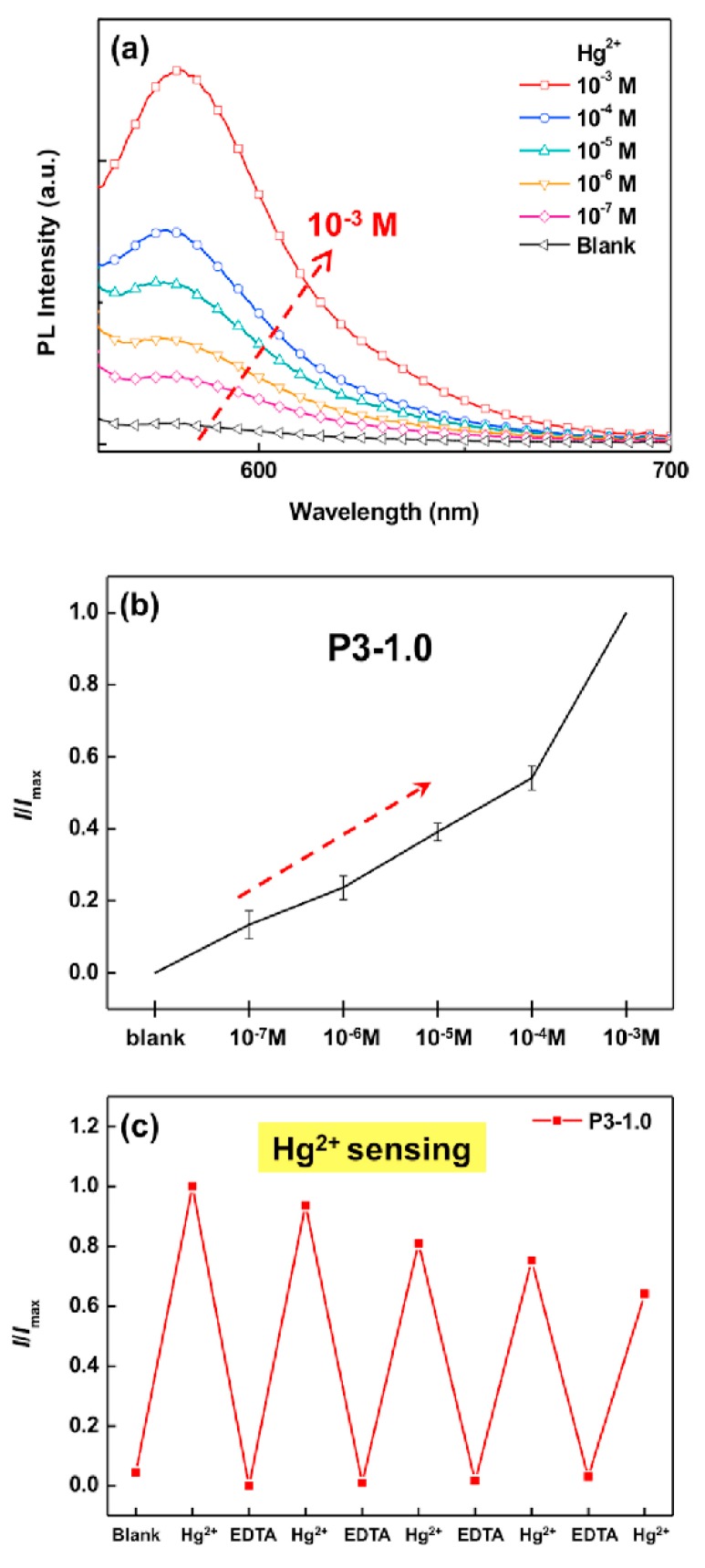
Spectra of ES nanofibers: (**a**) Variation in the PL spectra of P3-1.0 ES nanofibers in aqueous solutions with different molarities of Hg^2+^ ions from 0 M to 10^−3^ M. (**b**) Relative trend graph (*I/I_max_*) of P3-1.0 ES nanofibers. (**c**) Reversibility of the Hg^2+^-dependent “on–off–on” fluorescence intensity profile of P3-1.0 ES nanofibers (λ_ex_: 510 nm). Reprinted with permission from [41]. Copyright 2017, Elsevier.

Chen et al. fabricated the electrospun nanofiber-based fluorescent chemosensory filter membranes using poly(2-hydroxyethyl methacrylate-*co*-N-methylolacrylamide-*co*-rhodamine derivative) (poly(HEMA-*co*-NMA-*co*-RhBN2AM)) copolymers [41]. In short, the fluorescent probe N-(2-(3′,6′-bis(diethylamino)-3-oxospiro[isoindoline-1,9′-xanthen]-2-yl)ethyl)methacrylamide (RhBN2AM) was prepared first, and that was followed by the synthesis of poly(HEMA-co-NMA) with three different NMA ratios (P1, P2, and P3) using the free-radical polymerization. Afterward, the poly(HEMA-*co*-NMA-*co*-RhBN2AM) was synthesized with different ratios of RhBN2AM and the resultant products were named as P3-0.1, P3-0.5, and P3-1.0, which were further used for the preparation of electrospun nanofibers, and then chemical cross-linking was done through the annealing process. The HEMA was used for exhibiting the hydrophilic properties for absorption of Hg^2+^ ions in water, whereas the NMA moieties were chosen for the chemical cross-linking to maintain the fiber structure in water. The capability of the metal ion sensing was determined using the poly(HEMA-co-NMA-co-RhBN2AM) (P3-1.0) electrospun nanofibers prepared with 83.9:10.6:5.5 HEMA:NMA:RhBN2AM ratio, because their beneficial characteristics and Figure 4 depict the corresponding sensing response. The photoluminescence (PL) spectra of the P3-1.0 electrospun nanofibers after treatment with various concentrations of Hg^2+^ is presented in the Figure 4a and the results show that the intensity at 581 nm for the 10^−3^ M Hg^2+^ was higher than that of other concentrations, owing to a higher recognition of the Hg^2+^ ions by RhBN2AM in the electrospun nanofibers. The nonfluorescent spirocyclic form of the RhBN2AM turned to opened ring form following interaction with the Hg^2+^ ions, resulting in an increase of the fluorescence intensity, as depicted in the Figure 4b, and the limit of detection was found to be 10^−7^ M. Further, the reversibility of the sensor was evaluated through the ethylenediaminetetraacetic acid (EDTA) treatment and the results indicated that the emission was restored to the original state that leads to a Hg^2+^-dependent “on–off–on” fluorescence profile (see Figure 4c). Overall, the results indicate that the electrospun nanofibrous membrane possesses on/off switching capacity that could be applied in the heavy metal ion detection.

**Figure 5:**
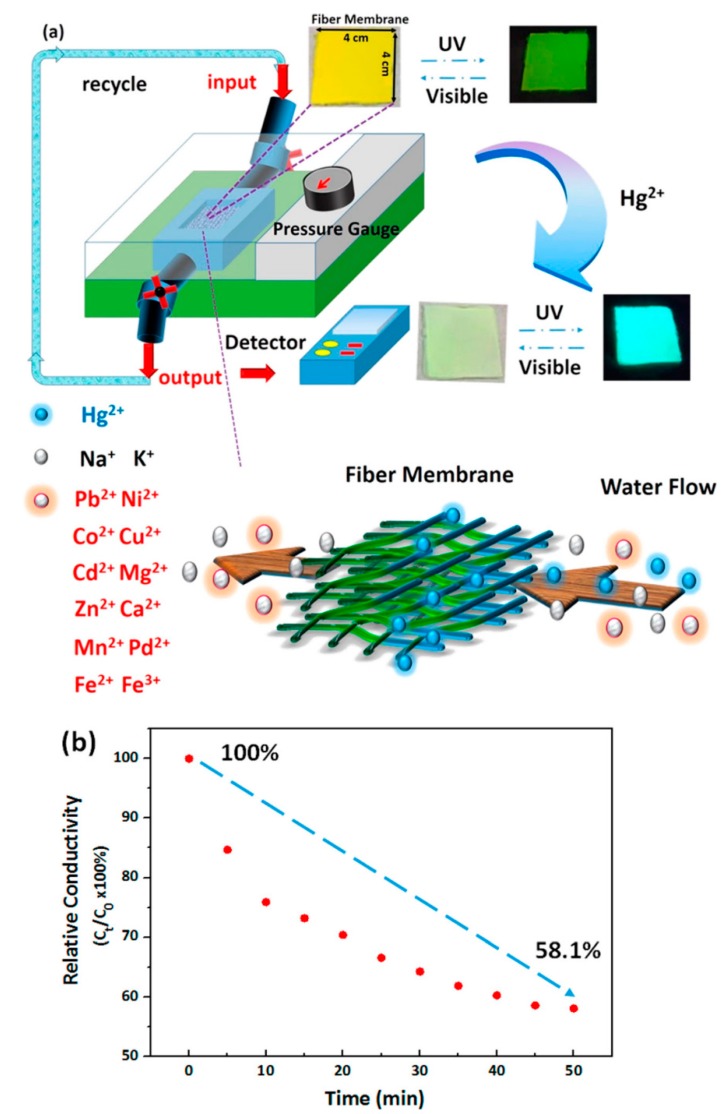
(**a**) Schematic illustration of a probe filter microfluidics system for real-time metal-ion sensing using an electrospun nanofiber membrane. (**b**) Relative conductivity versus time of the prepared Hg^2+^ solution in the microfluidics system. Reprinted with permission from [42]. Copyright 2017, American Chemical Society.

Similarly, fluorescent, porous, electrospun nanofibers-based on a novel red-green-blue (RGB) switchable probe with high sensitivity towards the pH and Hg^2+^ ions were prepared using a copolymer (poly(methyl methacrylatete-*co*-1,8-naphthalimide derivatives-*co*-rhodamine derivative); poly(MMA-*co*-BNPTU-*co*-RhBAM)) through a single-capillary spinneret [42]. The mechanism of the fluorescence detection for the Hg^2+^ was examined through the Förster resonance energy transfer (FRET). A similar composition was used for the preparation of the thin film using a drop casting method and the porous nanofibers were found to have a higher surface-to-volume ratio. Several techniques wereadopted to understand the morphology, chemical structures, and functional features of the moieties synthesized, and the functionalized electrospun nanofibers. Following systematic characterizations, the nanofibrous membrane prepared at the molar ratio 92.5:3:4.5 (P5) of MMA/BN/Rh was selected for the sensing experiments. In the initial phase of the sensing experiment, the response of the P5 electrospun nanofibers at different pH levels was investigated. The observations indicated that the exposure of Hg^2+^ at 10^–3^ M concentration leads to a blue shift in the emission spectra attributed to the thiourea unit of BNPTU transforming the imidazoline moiety, which resulted in a significant decrease of electron delocalization with in the fluorophore. Further, the comparative sensing performance between the P5 electrospun nanofibers and P5 thin film was monitored in neutral conditions at various concentrations of the Hg^2+^ ions. In both cases, the Hg^2+^ concentrations induced blue shift in the emission spectrum from 510 to 460 nm, and furthermore, the influence on the intensity depended on the concentration. The electrospun nanofibers have shown an improved sensitivity to the thin film, with a lowest detection limits of 10^–7^ M and 10^–5^ M, respectively. Next, the selectivity of the nanofibers was investigated by monitoring the response towards other competitive metal ions and the outcome revealed that the electrospun nanofiber behaves distinctly towards the Hg^2+^ ions. Based on those findings and advantages of the electrospun nanofibers, a microfluidics system was developed to measure the solution conductivity intended for real time sensing applications. Concisely, the microfluidic system was constructed by placing the electrospun nanofibrous web of 16 cm^2^ in middle of a tube to rapidly adsorb and sense the Hg^2+^ ions in a solution flow. As shown in Figure 5, the solution conductivity decreased from 132.7 μS cm^–1^ to 77.1 μS cm^–1^ when the exposure time increased from 0 to 50 min due to higher amount of adsorption resulting in less Hg^2+^ ions present in the solution. Interestingly, the electrospun nanofibers specifically adsorbed the Hg^2+^ in the mixed metal ion solutions, demonstrating their potential for the real-time, selective sensing of Hg^2+^ ions. Likewise, poly(N-Isopropylacrylamide-*co*-N-methylolacrylamide-*co*-rhodamine derivative) (poly(NIPAAm-*co*-NMA-*co*-RhBN2AM)) has been used for the fabrication of multifunctional electrospun nanofiber-based chemosensors for the sensing of Hg^2+^ ions [43].

**Figure 6:**
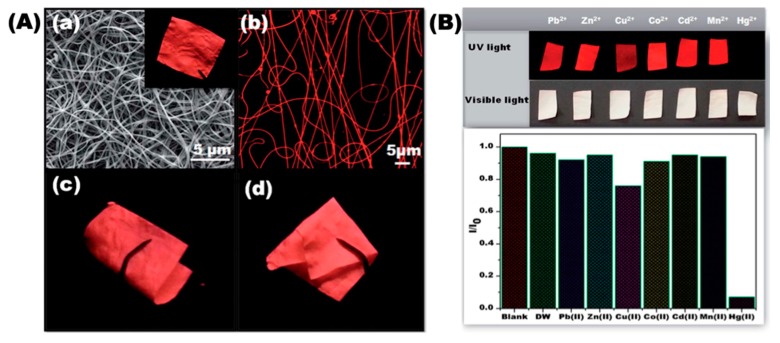
(**A**) SEM image of the cross-linked AuNC*NFM (**a**). Inset shows a photograph taken under UV light. (**b**) CLSM image of the AuNC*NF. (**c**,**d**) Flexible nature of the nanofibrous membrane. (**B**) Sensing performance of AuNC*NFM upon exposure to different metal ions in water. The concentration of all metal ions was fixed at 10 ppm. Photographs were taken under UV and white light. Reprinted with permission from [44]. Copyright 2014, The Royal Society of Chemistry.

In continuation to the progress of developing nanofiber based Hg^2+^ sensor, Senthamizhan et al. fabricated a polyvinyl alcohol (PVA) nanofibrous membrane (NFM) embedded with highly-luminescent gold nanoclusters (AuNC) named AuNC*NFM for Hg^2+^ detection [44]. The nanofibrous sensor strip was designed based on certain criteria, including (1) fine homogeneity of AuNC in the nanofibers, (2) stability across time and temperature, (3) insoluble nature in water, (4) specific response to Hg^2+^, and (5) significant visual colorimetric detection. The PVA polymer matrix was selected for its hydrophilicity, electrospinnability, compatibility, and nontoxicity characteristics. The PVA nanofibrous mat was cross-linked with the glutaraldehyde (GA) vapor to convert the mat to being water-insoluble. The AuNC*NFM demonstrated excellent long-term stability and preserved its fluorescence characteristics up to 100 °C, which does not affect the sensing performance during real-time application. Figure 6A demonstrates the morphology, fluorescent characteristics, and flexible nature of the AuNC*NFM. The contact mode approach was employed for the visual detection of Hg^2+^ through exposure to various concentrations, and the results demonstrate that the composite nanofibers are capable of detecting Hg^2+^ with a detection limit of 1 ppb. Since the nanofibers possess a larger surface area, which facilitates the rapid adsorption of the Hg^2+^ ions—that leads to desorption of capping molecules (bovine serum albumin, BSA) from the surface of AuNC, resulting in a color change from red to blue. Further, selectivity of the AuNC*NFM over competing chemical interferences was investigated by monitoring the responses towards Pb^2+^, Cd^2+^, Mg^2+^, Cu^2+^, Zn^2+^, and Co^2+^ at higher concentrations, and the corresponding finding is depicted in Figure 6B. The AuNC*NFM demonstrated no obvious color changes except for the Hg^2+^, but a slight decrease in the fluorescence was also noted for the Cu^2+^ ions; however, no significant quenching was noted when the concentration went down. The capability of visual color change, selectivity, sensitivity, and high stability of the AuNC*NFM offer trouble-free “naked eye” colorimetric sensing, and its utility in the real-time applications for monitoring toxic mercury in the environment.

Another study reported by Parsaee demonstrated the feasibility of using bio-synthesized gold nanoparticles as Hg^2+^ sensing probes [45]. The concentrated extracts of *Gracilaria canaliculata* alga were used as reductants for synthesizing the gold nanoparticles under ultrasonic conditions. The electrospun sensing probe was prepared from the solution mixture of gold nanoparticles, rhodamine B (RhB), and tetraethyl orthosilicate (TEOS) on a glass slide for evaluating the sensing performance. The chemosensor function can be explained based on the gold-amalgam formation, and the catalytic activity on sodium borohydride and RhB, which leads to a distinguishable change in color from red and yellow fluorescence to colorless by converting the amount of Hg^2+^ deposited on gold nanoparticles. The sensors showed the colorimetric and fluorescent detection limits of 2.21 nM and 1.10 nM, respectively. Furthermore, the sensor that was developed produced excellent performances in real-time samples, including tap and wastewater.

Recently, inorganic-organic, electrospun nanofibers have been fabricated by immobilization of carbazol-based Schiff base (S) into a polyvinyl alcohol (PVA)–TEOS polymeric support (PTSNFM), which has been used in the detection and removal of mercury (II) ions [46]. Shortly, as the first step, the Schiff base (S) was synthesized and then the PVA/TEOS/S nanofiber membrane (PTSNFM) was prepared from the precursor solution comprised of PVA/TEOS/S at a 16:12:1 ratio (wt%). The electrospun PTSNFM was dried in a vacuum oven for overnight, systematically characterized, and further used in the detection of Hg^2+^. The transmission electron microscopy (TEM) revealed that Schiff base nanoparticles (S-NPs) were capsulated both in/on the nanofibers. The fluorescence emission spectra recorded after treatment with Hg^2+^ ions showed an intense change from yellow to pink, whereas no such indications were noted with other metal ions, which confirmed the selectivity of PTSNFM. The limit of detection of the membrane was determined as of 0.0180 ng/mL; thus, the PTSNFM developed can be an excellent choice for the detection of mercury ions in aqueous media. However, the fabrication of sensors using this direct incorporation approach needs to address several factors, such as aggregation, stability over time/temperature/experimental process, quenching of fluorescence, and analyte diffusion into the nanofibers for their improved sensing performance.

### 2.2. Surface Decoration

**Figure 7:**
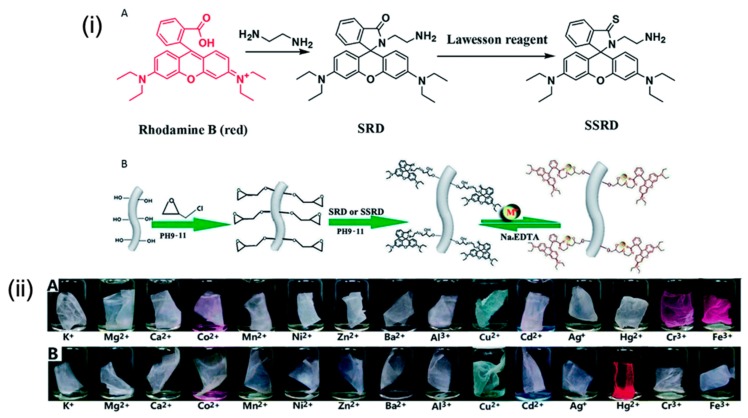
(**i**) Synthetic route of SRD and SSRD. (**A**) Schematic illustration of the functionalization of a poly(vinyl alcohol) electrospun nanofiber membrane by SRD (X = O, M*^n^*^+^ = Fe^3+^ or Cr^3+^) or SSRD (X = S, M*^n^*^+^ = Hg^2+^) and metal detection/adsorption applications (**B**). (**ii**) Photographs of the PVANF–SRD membrane (**A**) and PVANF–SSRD membrane (**B**) immersed into K^+^, Mg^2+^, Ca^2+^, Co^2+^, Mn^2+^, Ni^2+^, Zn^2+^, Ba^2+^, Al^3+^, Cu^2+^, Cd^2+^, Ag^+^, Hg^2+^, Cr^3+^, and Fe^3+^ aqueous solutions (1.0 × 10^−3^ M) for 5 min. Reprinted with permission from [50]. Copyright 2014, The Royal Society of Chemistry.

Several approaches have been developed for surface functionalization and employed in various applications, owing to the distinctive features of electrospun nanofibers [47,48,49]. Notably, the surface functionalization approach has a great effect in sensing applications, as large amounts of probes can be encapsulated because of the large surface area that results in enhanced interaction with analytes, which leads to more sensitive detection. To date, considerable progress has been made on designing and developing electrospun nanofibers through the surface functionalization approach for the sake of Hg^2+^ ion sensing. For instance, Wei et al. prepared poly(vinyl alcohol) electrospun nanofibrous (PVANF) membranes modified with spirolactam–rhodamine derivatives (PVANF–SRD) and sulfo-spirolactam–rhodamine derivatives (PVANF–SSRD) that sensitively detect metal ions [50]. Firstly, SRD and SSRD were synthesized and then functionalized in nanofibers, as shown in schematic Figure 7i. The PVA was dissolved and refluxed to obtain a homogeneous solution followed by the addition of glutaraldehyde (GA) prior to electrospinning. The resulting solution was further electrospun to obtain PVANF/GA nanofibrous membranes that were further immersed in a solution mixture containing 10% HCl and 90% methanol for 9 days in order to get a water-stable nanofibrous membrane. Further, the obtained membrane was subjected to immersion in a NaOH solution followed by the introduction of epichlorohydrin; the mixture was then placed on an incubating shaker, and afterward, thoroughly washed with distilled water. The membrane was then transferred and immersed in a mixture containing equal proportions of water and ethanol with SRD and SSRD, after which the mixture was placed in an incubator shaker, rinsed, and dried with nitrogen. The membranes were applied in the detection of metal ions. Precisely, the PVANF–SSRD membrane was employed for the detection of Hg^2+^ ions, whereas the PVANF–SRD membrane was used to detect Fe^3+^ and Cr^3+^ ions. As depicted in Figure 7iiB, the PVANF–SSRD membranes were immersed in the mercury and other ions solution at a concentration of 1 × 10^−3^ M, and the outcome revealed an excellent specific response to the Hg^2+^ ions. The exposure to various concentrations of Hg^2+^ ions indicated good sensitivity and selectivity, and a fast response with a detection limit of approximately 5.0 × 10^−7^ M. The membrane also showed good reversibility upon treatment with EDTA, indicating their sustainability in detection applications.

**Figure 8:**
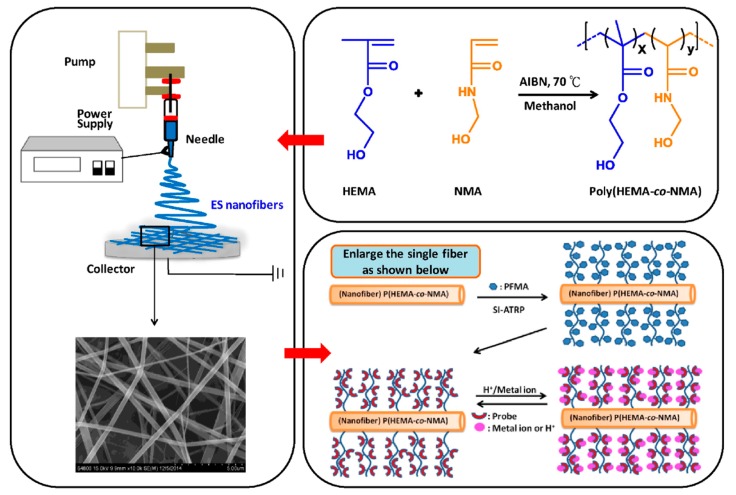
Design of multifunctional fluorescent ES nanofibers from poly(HEMA-*co*-NMA) ES nanofibers with grafted RhBN2 or SRhBOH. Reprinted with permission from [51]. Copyright 2016, Elsevier.

Cho et al. used pyrene derivative (PyDAN2) or rhodamine B derivative (RhBN2)–modified poly(2-hydroxyethyl methacrylate-*co*-N-methylolacrylamide) (poly(HEMA-*co*-NMA)) copolymer electrospun (ES) nanofibers for the detection of Cu^2 +^, Hg^2 +^, and pH [51]. The design and fabrication process of fluorescent electrospun nanofibers is presented in Figure 8. The study progressed with the combined efforts of synthesis, electrospinning, immobilization, characterization, and sensor application. Experimentally, a free radical polymerization process was used for the preparation of poly(HEMA*-co-*NMA), and then Hg^2 +^ sensing probe rhodamine derivatives were synthesized. The copolymer was used for the fabrication of nanofibers using electrospinning followed by crosslinking to enhance the stability, and then it underwent a series of processes to immobilize the functional probe on the surface in three steps. Briefly, initially the fibers were freeze dried and immobilized with 2-bromoisobutyryl bromide with the hydroxyl groups on the fiber surface (Fiber-Br) followed by surface grafting of pentafluorophenyl methacrylate amine-containing spirolactam (PPFMA) for surface-initiated atom transfer radical polymerization (SI-ATRP). As the final step, a mercury-responsive probe with RhBN2 was incorporated on the surfaces of the fibers, which was denoted as Fiber-*g*-RhBN2 and used in the detection of Hg^2 +^. The experimental sensor results showed an excellent selectivity toward Hg^2+^ rather than the other ions tested, including Cu^2+^, Cd^2+^, Cd^3+^, Fe^2+^, K^+^, Mg^2+^, Na^+^, Pb^2+^, and Zn^2 +^ through a substantial enhancement in PL intensity. The non-fluorescent RhBN2 transformed into ring open cyclic form, which led to strong fluorescence of an orange–red color upon interaction with Hg^2 +^. The Fiber-*g*-RhBN2 was found to have the minimum and maximum limits of detection at 10^−7^–10^−6^ M and 10^−2^–10^−1^ M, respectively, and the fluorescence was reversed upon treatment with EDTA, which can be used for at least four cycles. The outcome of the study indicated that the high surface-to-volume ratio of electrospun nanofibrous membranes plays a key role in the findings and that they can be used as “naked eye” sensors in environmental pollution monitoring.

**Figure 9:**
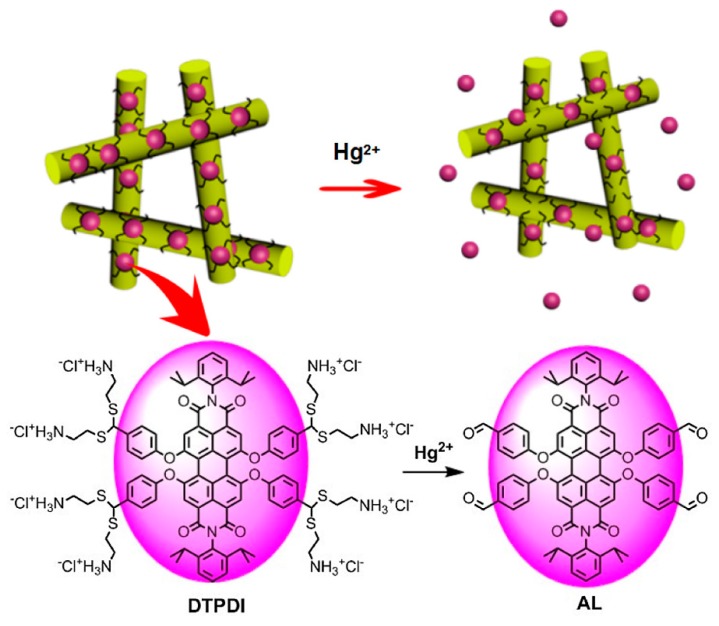
Schematic illustration for the sensing of Hg^2+^ by a fluorescent nanofibrous membrane (FNFM). Reprinted with permission from [52]. Copyright 2017, Elsevier.

Another study reported the preparation of a highly sensitive and selective fluorescent nanofibrous membrane (FNFM) using polyacrylonitrile (PAN) nanofibers and subsequent surface immobilization of dithioacetal-modified perylenediimide (DTPDI) for the detection of mercuric ions (II) that possess excellent stability under mechanical force through electrostatic interaction [52]. In order to prepare the FNFM, as the first step, the PAN nanofibers were prepared by electrospinning from the precursor solution of PAN dissolved in dimethylformamide (DMF). Then the PAN nanofibers were hydrolyzed with NaOH for the sake of achieving a negatively charged surface, and were washed with deionized water, which was followed by immersion in the DTPDI solution. That was further placed in a thermostatic shaker bath, washed, and dried. The resultant membrane was subsequently used in the sensing of Hg^2 +^ through treatment with several concentrations ranging from 1 ppm to 1 ppb. The outcome of the sensing experiment indicated that the FNFM has the potential to detect Hg^2 +^ as low as 1 ppb and that the detection limit meets the requirements set by World Health Organization (6 ppb) and the U.S. Environmental Protection Agency (2 ppb). Besides, the FNFM exhibited a selective response to Hg^2+^ over other co-existent ions. The sensing mechanism of the FNFM can be explained this way: upon interaction with Hg^2+^, C–S–C bonds present in the DTPDI were broken and produced oil-soluble AL dye, as depicted in Figure 9. Further, the durability of the FNFM was investigated by washing the FNFM with dichloromethane, and the results indicated that the performance was stable even after seven cycles.

**Figure 10:**
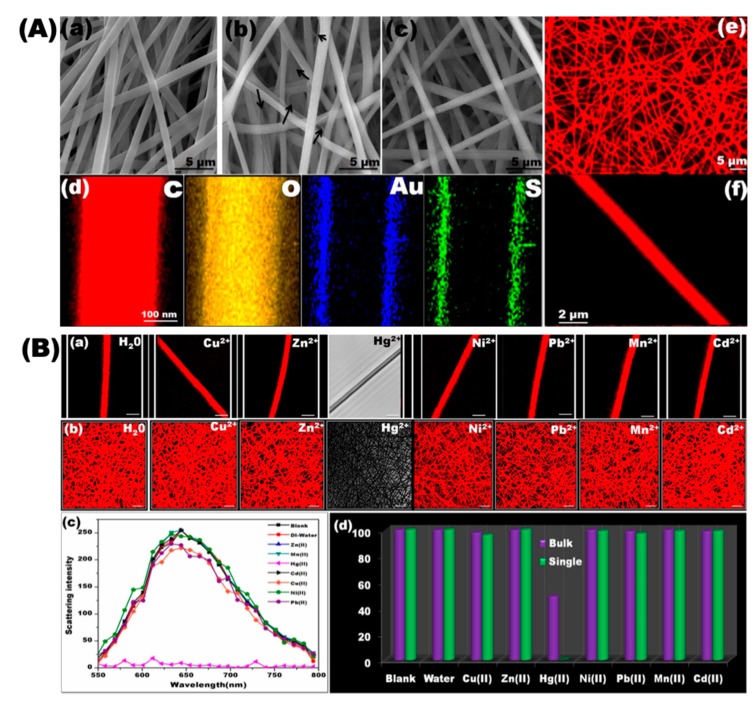
(**A**) Representative SEM images of the electrospun PCL-NF (**a**) gold nanocluster (AuNC) coated PCL-NF (AuNC*PCL-NF) in the presence (**b**) and absence (**c**) of excess BSA ligand. The arrow indicates the excess amount of weakly-adsorbed ligand across the NF surface. (**d**) HAADF-STEM elemental mapping of the C, O, S, and Au elements present in the AuNC*PCL single NF, shows AuNCs are consistently anchored on the surface of NFs. (**e**) Fluorescence image of AuNC*PCL-NF taken using a CLSM, excited at 488 nm. The red color emitted is owed to the characteristic emission of AuNC. (**f**) CLSM image of the AuNC*PCL single NF confirms the uniform bright fluorescent feature throughout the NF surface. (**B**) Selective sensing performance of AuNC*PCL-SNF and the AuNC*PCL-NF mat. The CLSM image presents the fluorescence response of AuNC*PCL-SNF (**a**) and the AuNC*PCL-NF mat (**b**) to various metal ions (indicated in each image) at concentrations of 10 ppm. (Scale bar (**a**)—2 μm, and (**b**)—5 μm; note: for Hg^2+^ only, DIC images are given since fluorescence is completely quenched and CLSM images become fully black.) The H_2_O treated AuNC*PCL-NFs show their stability and proves that the decreased fluorescence upon the addition of metal ions is not because of solvent. (**c**) Variation in the emission spectra of different metal ions treated with AuNC*PCL-SNF. (**d**) Bar diagram illustrating the relative variations in the fluorescence intensity of the single NF and nanofibrous mat. Reprinted from [53] 2015, Springer Nature Publishing AG.

To extend credit for designing and developing colorimetric sensors with enhanced Hg^2+^ detection, Senthamizhan et al. demonstrated real-time selective visual monitoring of Hg^2+^ at parts per trillion (ppt) level [53]. For this purpose, bovine serum albumin (BSA)-capped, fluorescent, gold nanoclusters (AuNC) were first synthesized and their emission characteristics were analyzed under a UV lamp. The observation indicated that the AuNC emitted a red color. Afterward, polycaprolactone (PCL) nanofibers (PCL-NF) were prepared from the precursor solution containing 18% (w/v) PCL polymer in a solvent mixture (3:1, v/v) of dimethylformamide (DMF) and dicholoromethane (DCM) by using electrospinning, and the nanofibers obtained were kept in vacuum oven to remove the residual solvent. A few seconds of the electrospinning process was performed in order to collect a single nanofiber (SNF). In the next phase, the fluorescent BSA capped AuNC was anchored on the surface of the PCL-NF by immersing it in the AuNC solution. Based on several characterization studies, the optimal time for coating and extraction of excessive ligands was fixed, since those parameters have a great influence on the interaction with Hg^2+^ and interfere with subsequent fluorescent characteristics. Figure 10A exemplifies the morphological, fluorescent, and elemental characteristics of the PCL-NF and gold nanocluster (AuNC)-coated PCL-NF termed AuNC*PCL-NF. The AuNC*PCL-NF possesses remarkable stability after more than four months in ambient conditions. The sensing experiments were carried out by dropping the desired concentration of metal ion solution on the nanofiber surface, and confocal laser scanning microscope (CLSM) images were taken after drying the nanofibers. In the real-time visual monitoring, the images were captured immediately following the metal ion solution passing, and corresponding spectra were recorded as a function of time. The sensing performance of AuNC*PCL-NF following exposure to various concentrations (50 ppt to 20 ppm) of Hg^2+^ revealed significant gradual decreases in fluorescence intensity upon increasing concentration with a detection limit of 50 ppt. At the higher concentrations (ppm), the fluorescence spectra were blue shifted, which might be attributable to the strong interaction between mercury and gold. Furthermore, the sensing experiments with tap water also exhibited similar responses, indicating their potential in practical applications. The selectivity of AuNC*PCL-NF and AuNC*PCL-SNF toward Hg^2+^ over various competing metal ions (Cu^2+^, Ni^2+^, Mn^2+^, Zn^2+^, Cd^2+^, and Pb^2+^) and the corresponding observations are presented in Figure 10B. There was a significant disappearance of fluorescence after exposure to Hg^2+^, while no apparent changes with other metal ions were noted. For the sake of extending the sensing application in real-time, the responses of AuNC*PCL-NF and AuNC*PCL-SNF were observed as a function of time following exposure to Hg^2+^ ions, resulting in a gradual fluorescent intensity decrease for increasing time. Interestingly, the AuNC*PCL-SNF demonstrated a faster response compared to bulk nanofibers. The sensing performance and selectivity were due to the improved interaction of gold and mercury that resulted in rapid adsorption; thus, leading to the formation of the Au–Hg amalgam, which has been systematically confirmed. Overall, stability, easy functionalization, and enhanced performance indicated that the sensor could be applied in real-time applications.

## 3. Summary and Future Directions

To sum up, as discussed throughout this review, the unique features of electrospun nanofibers facilitated good advancements in the designing and development of colorimetric sensor platforms for the effective detection of mercury ions. Accordingly, several probes have been functionalized in different varieties of electrospun nanofibers through direct incorporation and surface decoration approaches to invent colorimetric sensors with competitive performances. However, comparatively few efforts were made in embracing electrospun nanofibers for the colorimetric detection of mercury ions compared to the solution state. Moreover, the majority of the investigations focused on single metal ion detection, and huge challenges remain for the detection of multiple toxic metal ions using electrospun nanofiber-based sensor strip arrays. Therefore, concrete future efforts are needed for coupling the beneficial features of electrospinning and functional probes to develop electrospunnanofiber-based colorimetric sensors intended for real-time, on-site practical applications.

## References

[1] Järup L. (2003). Hazards of heavy metal contamination. Br. Med. Bull..

[2] Valko M., Morris H., Cronin M.T. (2005). Metals, toxicity and oxidative stress. Curr. Med. Chem..

[3] Rehman K., Fatima F., Waheed I., Akash M.S.H. (2018). Prevalence of exposure of heavy metals and their impact on health consequences. J. Cell. Biochem..

[4] Jaishankar M., Tseten T., Anbalagan N., Mathew B.B., Beeregowda K.N. (2014). Toxicity, mechanism and health effects of some heavy metals. Interdiscip. Toxicol..

[5] Ali H., Khan E., Ilahi I. (2019). Environmental chemistry and ecotoxicology of hazardous heavy metals: Environmental persistence, toxicity, and bioaccumulation. J. Chem..

[6] Holmes P., James K.A., Levy L.S. (2009). Is low-level environmental mercury exposure of concern to human health?. Sci. Total Environ..

[7] Tchounwou P.B., Ayensu W.K., Ninashvili N., Sutton D. (2003). Environmental exposure to mercury and its toxicopathologic implications for public health. Environ. Toxicol..

[8] Bernhoft R.A. (2012). Mercury toxicity and treatment: A review of the literature. J. Environ. Public Health.

[9] Genchi G., Sinicropi M.S., Carocci A., Lauria G., Catalano A. (2017). Mercury exposure and heart diseases. Int. J. Environ. Res. Public Health.

[10] Driscoll C.T., Mason R.P., Chan H.M., Jacob D.J., Pirrone N. (2013). Mercury as a global pollutant: Sources, pathways, and effects. Environ. Sci. Technol..

[11] Gworek B., Bemowska-Kałabun O., Kijeńska M., Wrzosek-Jakubowska J. (2016). Mercury in marine and oceanic waters—A review. Water Air Soil Pollut..

[12] Ullrich S.M., Tanton T.W., Abdrashitova S.A. (2001). Mercury in the aquatic environment: A review of factors affecting methylation. Crit. Rev. Environ. Sci. Technol..

[13] Poulain A.J., Garcia E., Amyot M., Campbell P.G.C., Raofie F., Ariya P.A. (2007). Biological and chemical redox transformations of mercury in fresh and salt waters of the high arctic during spring and summer. Environ. Sci. Technol..

[14] Made M., Yin Y., Zhang D., Liu J. (2016). Methods and recent advances in speciation analysis of mercury chemical species in environmental samples: A review. Chem. Speciation Bioavailability.

[15] Sarfo D.K., Sivanesan A., Izake E.L., Ayoko G.A. (2017). Rapid detection of mercury contamination in water by surface enhanced Raman spectroscopy. RSC Adv..

[16] Aragay G., Pons J., Merkoçi A. (2011). Recent trends in macro-, micro-, and nanomaterial-based tools and strategies for heavy-metal detection. Chem. Rev..

[17] Chansuvarn W., Tuntulani T., Imyim A. (2015). Colorimetric detection of mercury(II) based on gold nanoparticles, fluorescent gold nanoclusters and other gold-based nanomaterials. Trends Anal. Chem..

[18] Du J., Jiang L., Shao Q., Liu X., Marks R.S., Ma J., Chen X. (2013). Colorimetric detection of mercury ions based on plasmonic nanoparticles. Small.

[19] Xu X., Li Y.F., Zhao J., Li Y., Lin J., Li B., Gao Y., Chen C. (2015). Nanomaterial-based approaches for the detection and speciation of mercury. Analyst.

[20] Chen G., Guo Z., Zeng G., Tang L. (2015). Fluorescent and colorimetric sensors for environmental mercury detection. Analyst.

[21] Pokhrel L.R., Ettore N., Jacobs Z.L., Zarr A., Weir M.H., Scheuerman P.R., Kanel S.R., Dubey B. (2017). Novel carbon nanotube (CNT)-based ultrasensitive sensors for trace mercury(II) detection in water: A review. Sci. Total Environ..

[22] De Acha N., Elosúa C., Corres J.M., Arregui F.J. (2019). Fluorescent sensors for the detection of heavy metal ions in aqueous media. Sensors.

[23] Ding B., Yu J. (2014). Electrospun Nanofibers for Energy and Environmental Applications.

[24] Bagherzadeh R., Gorji M., Sorayani Bafgi M.S., Saveh-Shemshaki N., Afshari M. (2017). Electrospun conductive nanofibers for electronics. Electrospun Nanofibers.

[25] Senthamizhan A., Balusamy B., Aytac Z., Uyar T. (2016). Grain boundary engineering in electrospun ZnO nanostructures as promising photocatalysts. CrystEngComm.

[26] Anitha S., Brabu B., Thiruvadigal D.J., Gopalakrishnan C., Natarajan T.S. (2012). Preparation of free-standing electrospun composite ZnO membrane for antibacterial applications. Adv. Sci. Lett..

[27] Macagnano A., Zampetti E., Kny E. (2015). Electrospinning for High Performance Sensors.

[28] Anitha S., Brabu B., Rajesh K.P., Natarajan T.S. (2013). Fabrication of UV sensor based on electrospun composite fibers. Mater. Lett..

[29] Balusamy B., Sarioglu O.F., Senthamizhan A., Uyar T. (2019). Rational design and development of electrospun nanofibrous biohybrid composites. ACS Appl. Bio Mater..

[30] Focarete M.L., Gualandi C., Ramakrishna S. (2018). Filtering Media by Electrospinning: Next Generation Membranes for Separation Applications.

[31] Uyar T., Kny E. (2017). Electrospun Materials for Tissue Engineering and Biomedical Applications: Research, Design and Commercialization.

[32] Balusamy B., Senthamizhan A., Uyar T., Uyar T., Kny E. (2017). Electrospun nanofibrous materials for wound healing applications. Electrospun Materials for Tissue Engineering and Biomedical Applications: Research, Design and Commercialization.

[33] Ismail A.F., Hilal N., Jaafar J., Wright C. (2019). Nanofiber Membranes for Medical, Environmental, and Energy Applications.

[34] Senthamizhan A., Balusamy B., Uyar T. (2020). Recent progress on designing electrospun nanofibers for colorimetric biosensing applications. Curr. Opin. Biomed. Eng..

[35] Senthamizhan A., Balusamy B., Uyar T. (2016). Glucose sensors based on electrospun nanofibers: A review. Anal. Bioanal. Chem..

[36] Zhang N., Qiao R., Su J., Yan J., Xie Z., Qiao Y., Wang X., Zhong J. (2017). Recent advances of electrospun nanofibrous membranes in the development of chemosensors for heavy metal detection. Small.

[37] Terra I.A.A., Mercante L.A., Andre R.S., Correa D.S. (2017). Fluorescent and colorimetric electrospun nanofibers for heavy-metal sensing. Biosensors.

[38] Sahay R., Kumar P.S., Sridhar R., Sundaramurthy J., Venugopal J., Mhaisalkar S.G., Ramakrishna S. (2012). Electrospun composite nanofibers and their multifaceted applications. J. Mater. Chem..

[39] Kacmaz S., Ertekin K., Suslu A., Ergun Y., Celik E., Cocen U. (2012). Sub-nanomolar sensing of ionic mercury with polymeric electrospun nanofibers. Mater. Chem. Phys..

[40] Si Y., Wang X., Li Y., Chen K., Wang J., Yu J., Wang H., Ding B. (2014). Optimized colorimetric sensor strip for mercury(II) assay using hierarchical nanostructured conjugated polymers. J. Mater. Chem. A.

[41] Chen B.Y., Kuo C.C., Cho C.J., Liang F.C., Jeng R.J. (2017). Novel fluorescent chemosensory filter membranes composed of electrospun nanofibers with ultra-selective and reversible pH and Hg^2+^ sensing characteristics. Dyes Pigment..

[42] Liang F.C., Kuo C.C., Chen B.Y., Cho C.J., Hung C.C., Chen W.C., Borsali R. (2017). RGB-switchable porous electrospun nanofiber chemoprobe-filter prepared from multifunctional copolymers for versatile sensing of pH and heavy metals. ACS Appl. Mater. Interfaces.

[43] Chen B.Y., Lung Y.C., Kuo C.C., Liang F.C., Tsai T.L., Jiang D.H., Satoh T., Jeng R.J. (2018). Novel Multifunctional luminescent electrospun fluorescent nanofiber chemosensor-filters and their versatile sensing of pH, temperature, and metal ions. Polymers.

[44] Senthamizhan A., Celebioglu A., Uyar T. (2014). Flexible and highly stable electrospun nanofibrous membrane incorporating gold nanoclusters as an efficient probe for visual colorimetric detection of Hg(II). J. Mater. Chem. A.

[45] Parsaee Z. (2018). Electrospun nanofibers decorated with bio-sonochemically synthesized gold nanoparticles as an ultrasensitive probe in amalgam-based mercury (II) detection system. Ultrason. Sonochem..

[46] Tahvili A., Poush M.K., Ahmed M., Parsaee Z. (2019). New efficient inorganic-organic nanofibers electrospun membrane for fluorescence detection and removal of mercury (II) ions. J. Mol. Struct..

[47] Yoo H.S., Kim T.G., Park T.G. (2009). Surface-functionalized electrospun nanofibers for tissue engineering and drug delivery. Adv. Drug Deliv. Rev..

[48] Balusamy B., Senthamizhan A., Uyar T., Ismail A.F., Hilal N., Jaafar J., Wright C. (2019). Surface functionalized electrospun nanofibers for removal of toxic pollutants in water. Nanofiber Membranes for Medical, Environmental, and Energy Applications.

[49] Senthamizhan A., Balusamy B., Aytac Z., Uyar T. (2016). Ultrasensitive electrospun fluorescent nanofibrous membrane for rapid visual colorimetric detection of H_2_O_2_. Anal. Bioanal. Chem..

[50] Wei Z., Zhao H., Zhang J., Deng L., Wu S., He J., Dong A. (2014). Poly(vinyl alcohol) electrospun nanofibrous membrane modified with spirolactam–rhodamine derivatives for visible detection and removal of metal ions. RSC Adv..

[51] Cho C.J., Lu S.T., Kuo C.C., Liang F.C., Chen B.Y., Chu C.C. (2016). Pyrene or rhodamine derivative–modified surfaces of electrospun nanofibrous chemosensors for colorimetric and fluorescent determination of Cu^2+^, Hg^2+^, and pH. React. Funct. Polym..

[52] Ma L., Liu K., Yin M., Chang J., Geng Y., Pan K. (2017). Fluorescent nanofibrous membrane (FNFM) for the detection of mercuric ion (II) with high sensitivity and selectivity. Sens. Actuators B Chem..

[53] Senthamizhan A., Celebioglu A., Uyar T. (2015). Real-time selective visual monitoring of Hg^2+^ detection at ppt level: An approach to lighting electrospun nanofibers using gold nanoclusters. Sci. Rep..

